# NAFLD and cardiovascular disease

**DOI:** 10.1016/j.pbj.0000000000000002

**Published:** 2018-07-18

**Authors:** Elisabete Martins, Ana Oliveira

**Affiliations:** a Department of Medicine, Faculty of Medicine; b Instituto de Investigação e Inovação em Saúde (i3s), University of Porto; c Department of Cardiology; d Department of Nuclear Medicine, São João Hospital Center, Porto, Portugal.

**Keywords:** cardiovascular, hepatic steatosis, NAFLD, nonalcoholic fatty liver disease

## Abstract

Nonalcoholic fatty liver disease (NAFLD) is an important cause of chronic hepatic disease and liver transplant in Western societies. The increasing prevalence is related to dietary changes and sedentarism and follows the increasing frequency of obesity and type 2 diabetes mellitus.

Growing evidence of association of NAFLD with cardiovascular diseases (CVD), independent of cardiovascular risk factors, has prompted the clarification of whether the liver is mainly a key-effector or a target-organ of the metabolic disarrangements in the metabolic syndrome. The therapeutic strategies able to alter liver disease progression and, through this, reduce the cardiovascular risk have also been tested in the last 2 decades.

This review focus on the possible interactions between hepatic disease, metabolic syndrome, and CVD, and on their implications for clinical practice.

## Hepatic steatosis: Definitions, epidemiology, and diagnosis

Nonalcoholic fatty liver disease (NAFLD) is one of the most frequent causes of chronic liver disease in Western societies.^1^

NAFLD mimics the hepatic abnormalities caused by alcohol consumption in patients with a reduced (<30 g in men and 20 g in women) to null daily consumption of alcohol.^2^ It is a clinicopathological entity with a wide histological spectrum, starting at hepatic steatosis, defined as fat accumulation in at least 5% of hepatocytes.^3^ The natural history of NAFLD, though not established,^4^ can be considered in 4 stages: (1) “Simple” steatosis; (2) nonalcoholic steatohepatitis (NASH)—steatosis accompanied by inflammatory changes and “ballooning” (degeneration) of hepatocytes, eventually leading to necrosis; (3) cirrhosis (fibrosis and nodular changes [steatosis may disappear over time]); and (4) hepatocarcinoma (hepatocellular carcinoma, can occur in the absence of cirrhosis).

Risk factors for NAFLD progression (fibrosis) were age >45 to 50, the presence of type 2 diabetes mellitus (T2DM), the degree of insulin resistance, body mass index >28 to 30 kg/m^2^, hypertension,^5^ and genetic polymorphisms.^6^ The risk of progression associates with the risk of cirrhosis and hepatocarcinoma.

NAFLD frequency has progressively augmented,^7^ even in pediatric ages, due to diet changes, sedentary lifestyle, and the increasing prevalence of obesity and T2DM. Its true incidence and prevalence are unknown; estimates of prevalence range widely from 11% to 46%.^8,9^ In unselected populations, proton-magnetic resonance spectroscopy (^1^H-MRS) revealed hepatic steatosis in 33.6% of 2349 adults.^10^ In diabetics, NAFLD may affect up to 70% of the patients.^11,12^ NASH affects 3% to 5% of the population^13^ and 25% to 30% of patients with obesity or T2DM.^14^ NAFLD/NASH cirrhosis is expected to be the major underlying cause for liver transplantation in Western countries by 2020.^15^ In the presence of NASH or cirrhosis, the risk of hepatocarcinoma is 7% to 15%.

Commonly used criteria for the clinical diagnosis of NAFLD^16^ are the presence of steatosis—demonstrated by imaging or histology—and the absence of excessive alcohol consumption or other causes of steatosis—viruses,^17^ drugs,^18^ or autoimmunity.^19^

Liver biopsy remains the gold standard for the diagnosis of NAFLD, but results from new imaging methods, such as ^1^H-MRS,^20^ magnetic resonance imaging, or computed tomography, correlate well with histologically detected steatosis.^21^ Abdominal ultrasound is still the first-line imaging method in clinical practice, due to its low cost and good accessibility. However, ultrasonography is believed to be of limited sensitivity (60–90%) when less than a third of hepatocytes are steatotic.^22^ Liver biochemistry correlates poorly with the presence of NAFLD,^23^ although serum transaminases and γ-glutamyltransferase levels may be elevated.^24^

## NAFLD and CVD

Patients with NAFLD have a higher prevalence of clinical cardiovascular diseases (CVD) than control individuals without steatosis. Moreover, CVD are the leading cause of death in NAFLD.^25–31^

NAFLD is a risk factor for diabetes^32^ in obese and nonoverweight individuals.^33–35^ Also, compared to patients who do not have NAFLD, patients with T2DM and NAFLD often have poorer glycemic control, more microvascular complications^36^ and an increased risk of all-cause mortality.^37^

NAFLD and the metabolic syndrome (MetS) components overlap significantly and the main concurrence between the 2 is insulin resistance. Up to 90% of the patients with NAFLD present at least one of the features of MetS—elevated triglycerides, reduced high-density lipoprotein (HDL), and elevated fasting-glucose levels, elevated waist circumference, and elevated blood pressure. One-third meets the MetS diagnosis criteria.^38,39^

Although the causal association between NAFLD and CVD is controversial,^26,28,40,41^ the evidence of an independent contribution of hepatic steatosis to clinical coronary heart disease and increased risk of fatal and nonfatal cardiovascular events (in both diabetic and nondiabetic) was provided by more than 20 studies, including meta-analyses.^25,29,32,42–46^ In those studies, diagnosis of the hepatic disease was made using different methodologies but mostly with ultrasonography.

NAFLD is also associated with subclinical atherosclerosis independently of conventional cardiovascular risk factors.^47^ A meta-analysis involving 3497 subjects confirmed the association of NAFLD with carotid intima-media thickness (IMT) and with an increased prevalence of carotid plaques.^48^ In the coronary territory, NAFLD was related to increased artery calcification scores^49–52^ (Table 1), increased severity of coronary stenosis,^53^ and abnormal coronary flow reserve.^54,55^

**Table 1:**
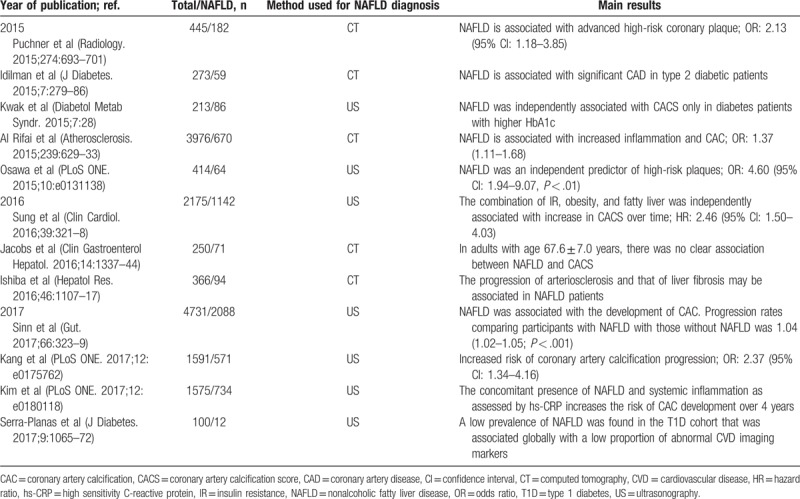
Recent studies (2015–2017) that evaluated the association between nonalcoholic fatty liver disease and coronary artery calcification

More uncertainties exist about the relation between the severity of hepatic disease and CVD risk.^30,31^ The presence of liver fibrosis was associated with cardiovascular organ damage (IMT and left ventricular hypertrophy)^56^ and increased mortality (hazard ratios of 3.46^57^ and 4.36^58^ for cardiovascular death) in patients with higher fibrosis scores.

Compared to those with simple steatosis, patients with NASH have more metabolic abnormalities,^59^ and an increasing risk of carotid disease^60^ and CVD mortality.^30,31,61^ By contrast, a recent meta-analysis found no significant difference, in terms of cardiovascular risk, between the presence of simple steatosis and NASH.^33^ Further studies in patients with biopsy-confirmed NAFLD are needed to clarify this issue.

Other NAFLD-related cardiac abnormalities that can explain higher cardiovascular risk include: changes of left ventricular structure and diastolic dysfunction,^62–66^ myocardial steatosis,^67^ aortic valve sclerosis,^68,69^ heart failure,^70,71^ atrial fibrillation,^72,73^ cardiac autonomic dysfunction,^74^ and prolonged heart rate-corrected QT interval.^75^ The pathophysiology of these associations is diverse and incompletely understood.

## Pathophysiological links

Regarding NAFLD and CVD, it is crucial to determine whether hepatic steatosis is an independent cardiovascular risk factor or a surrogate manifestation of a systemic atherogenic metabolic milieu—in particular, of insulin resistance. As a fundamental regulator of lipid and glucose metabolism, the liver can be the trigger and/or target of a metabolic syndrome.

**Figure 1:**
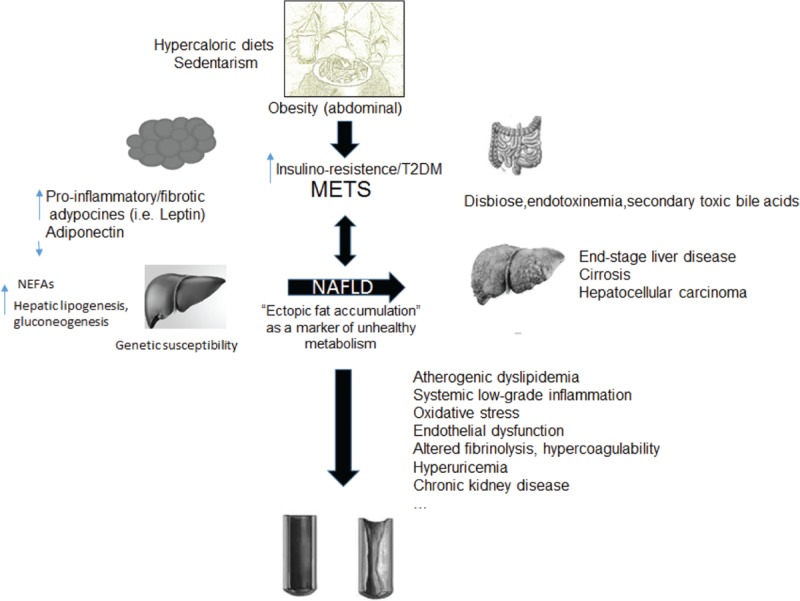
Proposed pathophysiological links between metabolic syndrome, nonalcoholic fatty liver disease, and cardiovascular disease. METS = metabolic syndrome, NAFLD = nonalcoholic fatty liver disease, NEFA = nonesterified fatty acids, T2DM = type 2 diabetes mellitus.

The mechanisms of NAFLD development are largely unknown, probably multifactorial and resulting from complex interactions between environmental and genetic factors. The contribution of parallel factors, acting synergistically in genetically predisposed individuals, is the basis for the “multiple-hit” hypothesis for NAFLD development and progression^76^ (Fig. 1).

Environmental factors include hypercaloric diets (particularly those rich in saturated fats, refined carbohydrates, and high fructose-sweeteners)^77^ and sedentarism. Both are associated with obesity (abdominal), metabolic syndrome, and NAFLD.

The hallmark of NAFLD is triglyceride accumulation in the cytoplasm of hepatocytes due to an imbalance between lipid acquisition (ie, fatty acid uptake and *de novo* lipogenesis) and lipid removal (ie, mitochondrial fatty acid oxidation and export as a component of very low density lipoproteins [VLDL] particles).^78^ The excessive caloric intake results in a spillover of nonesterified fatty acids from adipose visceral tissue to ectopic fat storage (namely the liver), due to insufficient oxidation and VLDL secretion. Peripheral lipolysis in peripheral adipose tissue, aggravated by insulin resistance, is the source of most hepatic lipids but *de novo* lipogenesis also plays a substantial role in the pathogenesis of NAFLD.^79^

There is an important cross-talk between the liver and the expanded, ectopic and dysfunctional (inflamed) adipose tissue. This affects the metabolism of fatty acids, promoting the deposition of triglycerides in organs such as the liver itself. The adipose tissue significantly contributes to the systemic metabolic state, due to an unbalance between inflammatory and anti-inflammatory/antifibrotic adipocytokines. High tumor necrosis factor (TNF)-α and low adiponectin levels are associated with NAFLD, independently of insulin resistance.^80^

In turn, the accumulation of intrahepatic fat changes the insulin signal at the hepatic level, promoting gluconeogenesis, which then promotes hyperglycemia and increases the risk of T2DM.^81^ Liver damage caused by fat accumulation is due to several mechanisms including lipotoxicity, increased oxidative stress, endothelial dysfunction, apoptosis, and inflammation. Hepatic lipid accumulation leads to subacute hepatic inflammation via nuclear factor κB activation by releasing proinflammatory cytokines such as interleukin-6 (IL)-6, IL-1β, and TNF-α.

Pathologic liver angiogenesis has also been described in NAFLD^82^ although less prominent than in conditions of bridging or postnecrotic fibrosis (eg, in chronic viral infection). A connection between pathologic angiogenesis and fibrogenesis is suggested by experimental models of antiangiogenic therapy that are highly effective in reducing fibrogenic progression.

Leptin, an adipocytokine elevated in NAFLD patients, exerts profibrogenic effects during the progression from NAFLD to NASH and also operates as a proangiogenic mediator through the recruitment and stabilization of hypoxia-inducible factor 1-alpha (HIF-1α) and the nuclear translocation of HIF.

The liver is recognized as an endocrine organ that secretes hepatokines.^83^ These regulate systemic metabolism and energy homeostasis, and may contribute to the pathogenesis not only of NAFLD but also of metabolic syndrome, T2DM and CVD.

Some of the most recently recognized hepatokines involved in NAFLD pathogenesis are Fetuin-A, fibroblast growth factor 21 (FGF-21), selenoprotein P (SEPP1), sex hormone-binding globulin (SHBG), angiopoietin-related growth factor, and leukocyte cell-derived chemotaxin 2 (LECT2). Fetuin-A might constitute a link between obesity, insulin resistance, and NAFLD—it binds the insulin receptor in tissues, inhibiting insulin signaling and therefore inducing insulin resistance.^84,85^ Circulating FGF-21, also considered a metabolic regulator, significantly correlates with the hepatic fat content and could be regarded as a noninvasive biomarker useful in differentiating simple fatty liver from NASH.^86^ Serum LECT2 levels are also significantly higher in patients with obesity and NAFLD.^87^ On the contrary, serum concentrations of SHBG decreased with an increase of intrahepatic fat content.^88^

In patients with T2DM and with NAFLD alike, SEPP1, a selenium-carrier protein, correlated with cardiovascular risk factors (eg, subclinical parameters of inflammation and arterial stiffness).^89^ More recently, angiopoietin-like protein 8 (ANGPTL-8) was pointed as a predictor of significant NAFLD, independent of obesity and insulin resistance, and could also be a potential therapy target of NAFLD.^90^

Exploring the mechanisms of these and other hepatokines can determine their usefulness as biomarkers (of NAFLD, of disease progression or of CVD risk) or as primordial metabolic therapeutic targets. Further experimental and clinical studies are necessary to confirm these hypotheses.

Genetic factors also account for a significant percentage of hepatic fat variability.^91^ Single nucleotide polymorphisms in genes involved in lipid metabolism (Lipin 1, patatin-like phospholipase domain containing-3 [PNPLA3]), oxidative stress (superoxide dismutase 2), insulin signaling (insulin receptor substrate-1), and fibrinogenesis (Kruppel-like factor 6) are associated with a risk for the development and progression of NAFLD.^92^

Carriers of the genetic variant p.I148M (rs738409 C/G) in PNPLA3 gene have a higher risk of liver damage in the presence of external noxious, raised levels of aspartate aminotransferase and/or alanine aminotransferase,^93^ and more severe NASH with greater levels of fibrosis.^94,95^ PNPLA3 gene is recognized as a modifier in terms of NAFLD disease severity and risk of related hepatocarcinoma,^96^ but is not associated with insulin resistance^97^ and has apparently no significant effect on NAFLD-related CVD risk.^98–100^

The dissociation between genetic and metabolic-driven NAFLD in terms of CVD risk was also put in evidence in another study, where carriers of the transmembrane 6 superfamily member 2 (TM6SF2) E167K variant were more susceptible to progressive NASH, but were protected against CVD disease.^101^

In some cases, however, NAFLD may even arise due solely to genetic factors—in the absence of weight overload or metabolic syndrome.^33,102^ With time, the “genetic” NAFLD may evolve into a metabolic systemic disturbance associated with secondary insulin resistance.

The elevated incidence of CVD in the presence of hepatic disease may be due to the interaction of factors that cause or aggravate liver damage itself. The liver of patients with NAFLD might be the source of the proatherogenic, proinflammatory, and diabetogenic mediators.^103^ Contributing atherogenic factors in NAFLD are dyslipidemia characterized by high triglycerides, small and dense low-density lipoprotein, lower HDL, higher plasma apolipoprotein B to apolipoprotein A1 ratio, oxidative stress,^104^ altered biology of adipokines,^105^ genetic predisposition, chronic kidney disease, hypovitaminosis D_3_, hyperuricemia, increased procoagulants (fibrinogen, plasminogen activator inhibitor-1, factor VIII, transforming growth factor [TGF]), altered fibrinolysis, endothelial dysfunction, and chronic subclinical inflammation (elevation of IL-6, TNF-α, C-reactive protein, fibrinogen).

Considering hemostatic alterations, it has been shown that NAFLD patients display a procoagulant imbalance that progresses along with the severity of the disease (from steatosis/NASH to metabolic cirrhosis) and might be responsible for the increased risk of thrombosis and/or liver fibrosis observed in these patients.^106^

Changes of intestinal microbioma have also been associated with dietary changes, abdominal obesity, metabolic syndrome, chronic inflammation, and NAFLD.^107^ It is unknown if disbiose could independently affect the CVD risk of patients with NAFLD.

In NAFLD, the intricate interactions between liver and extra-hepatic organs, atherosclerotic risk factors, and CVD requires a multidisciplinary, personalized approach capable of reducing the risk of hepatic and nonhepatic morbidity and mortality.

## Cardiovascular risk: Diagnosis and management

### Screening measures and cardiovascular risk assessment

Early diagnosis of NAFLD with early therapeutic measures may reduce both liver-related complications and CVD. However, given the large number of individuals involved, it is difficult to implement screening strategies for hepatic disease. The best preventive measures include prevention and control of cardiovascular risk factors.^108,109^

In patients with NAFLD, cardiovascular risk stratification should be assessed, ideally calculating the global CVD risk in the individual patient, bearing in mind that the usual scoring systems may underestimate the degree of insulin resistance, hypertriglyceridemia, or subclinical inflammation.

The Framingham risk score, that determines the risk at 10 years of CVD events, appears to be sufficiently accurate in predicting risk in these patients.^110^ Timing for re-assessment, although poorly defined, should be determined depending on the baseline risk.^111^ Carotid IMT, coronary artery calcium score, or other diagnostic parameters may be useful for a more accurate reclassification of individuals at intermediate risk.^112^

### Lifestyle changes

Since NAFLD is mainly a manifestation of obesity and metabolic syndrome, lifestyle modification remains the cornerstone of prevention and treatment. Weight loss, diet, and regular exercise have all independent beneficial effects on hepatic disease.

Even modest losses of weight improve steatosis, inflammation, ballooning, and hepatic disease severity.^113^ A general objective is restricting calories intake, aiming for a weight loss of at least 5% to 10%.^114^

Bariatric surgery, reserved to patients with severe obesity, also improves hepatic steatosis,^115^ but rapid weight reduction should be avoided.^116,117^ Insufficient data are available regarding antiobesity therapies including orlistat, an inhibitor of pancreatic lipase.^118^

Concerning diet, there are few data on the effect of macronutrients. Adherence to the Mediterranean diet (with increased polyunsaturated fatty acids intake, reduced fat and carbohydrates intake, high fiber diet) has known beneficial effects,^119,120^ even in pediatric patients,^121^ as also the avoidance of fructose-sweeteners.^122^

The effect of alcohol in patients with NAFLD is still debated. Light-to-moderate alcohol consumption may exert some beneficial effect on the severity of NAFLD.^123,124^

Regular exercise contributes to increasing insulin sensitivity and abdominal fat reduction. There is an inverse relationship between exercise and NAFLD, independent of weight loss.^125–127^ The optimal physical activity regimen still remains to be determined. A recent trial showed that 4 months of resistance training and aerobic training were equally effective in patients with T2DM.^128^

### Pharmacological treatments

There are no pharmacological agents approved specifically for NAFLD treatment, so the main concern is patients with NASH, who have the higher risk of disease progression.

Considering known pathophysiologic links involved in NAFLD, different pharmacologic approaches have been tested; however, until now, there is a lack of large randomized controlled trials (RCT) reporting histological outcomes.

As insulin resistance is an essential requirement for the accumulation of hepatocellular fat, most studies have focused on the effects of antidiabetic drugs.

Metformin, used for decades for the treatment of T2DM, improves insulin resistance, but displays marginal effects on transaminases, with no improvement in steatosis or inflammation in NAFLD, in larger controlled clinical trials.^129^

By contrast, pioglitazone, a thiazolinedione which acts as a selective agonist of peroxisome proliferator-activated receptor (PPAR)-γ improving systemic and hepatic insulin sensitivity, is the drug with the highest level of evidence for the treatment of NASH in patients with prediabetes/T2DM, as it improved steatosis and inflammation and may reduce hepatic fibrosis.^130,131^

Another PPAR modulator, elafibranor (GFT505), combines PPAR-α effects, which primarily enhance lipid metabolism, with PPAR δ effects, which target more glucose homeostasis. In GOLDEN trial,^132^ it was tested in patients with biopsy-proven NASH with a primary histological end-point: NASH resolution without fibrosis worsening. This result was achieved in 23% and 21% of patients treated with 80 and 120 mg/d, respectively, leading to a large multicenter RCT in patients with more severe steatohepatitis.

Incretin mimetics, as glucagon-like peptide 1 (GLP-1) receptor agonists (exenatide and liraglutide), increase insulin sensitivity, lower postprandial glucagon levels, and induce weight loss.^133–135^ Liraglutide may improve hepatic steatosis,^136^ and in Liraglutide Efficacy and Action in Non-alcoholic steatohepatitis (LEAN) trial was able to resolve biopsy-proven NASH in 39% of the treated patients. However, it is still uncertain if the treatment benefit was caused by the weight reduction (responders had a mean weight loss of 2.1 kg).^137^ The drug should be tested further in larger studies with long follow-ups.^138^

Dipeptidyl peptidase-4 (DPP-4) inhibitors exert glucose-lowering effects primarily by blocking the enzyme DPP-4 that degrades GLP-1, and so enhances the endogenous level of GLP-1. Sitagliptin and vildagliptin reduce plasma aminotransferases^139,140^ and could ameliorate hepatic steatosis and inflammation,^141^ but limited clinical data are still available.

There are also no studies assessing the effect on NAFLD patients liver histology of Sodium–Glucose Co-Transporter 2 (SGLT2) Inhibitors (canagliflozin or dapagliflozin), agents that lower renal glucose absorption.

Beyond antidiabetics, other agents with different mechanisms of action have been tested in NAFLD, with different results.

Omega-3 polyunsaturated fatty acids (ω-3 PUFAs) have long been credited with reducing hepatic steatosis. Potential actions may be the regulation of hepatic gene expression, the improvement of insulin sensitivity, and the reduction of inflammation and of oxidative stress.^142^ The WELCOME Trial tested the effects of the regular ingestion of 4 g of eicosapentaenoic acid plus docosahexaenoic acid (approved for treatment of hypertriglyceridemia),^143^ and showed an improvement of NAFLD.^144^ However, other trials have been overall negative.^145,146^ It is also possible that particular PUFAs may be more beneficial than others.

Considering chronic oxidative stress as a key mechanism in liver damage and NAFLD progression, in PIVENS study vitamin E (α-tocopherol)—an antioxidant—was administered to nondiabetics, 800 IU/d for 96 weeks, resulting in improved steatosis, inflammation, and hepatocellular ballooning^147^; however, further evidence is necessary to support the efficacy of this fat-soluble agent for the treatment of NASH.

Considering the metabolic, anti-inflammatory and antifibrotic effects of vitamin D on hepatocytes^148^ and that NAFLD subjects are more likely to be vitamin D deficient, its use in NAFLD was tested in a small double-blind, placebo-control study. The obtained negative results in terms of steatosis markers warrant more studies to demonstrate the benefits with this vitamin supplementation.^149^

The use of acid obeticholic—a potent activator of the farnesoid X-nuclear receptor that regulates bile acid, glucose, and cholesterol homeostasis—also resulted in the improvement of steatosis, hepatocellular ballooning, and lobular inflammation in the FLINT trial.^150^ Compared to placebo, the NASH resolution was obtained in 22% of treated patients. One mechanism proposed to explain this benefit is the inhibition of intestinal cholesterol absorption by modulation of the bile acid pool, thus increasing reverse cholesterol transport.^151^ Long-term safety of this drug needs to be addressed and a phase III study is now ongoing.

Pentoxifylline inhibits a number of proinflammatory cytokines including TNF-α and may be hepatoprotective and antifibrogenic. In a randomized placebo-controlled trial, pentoxifylline improved histologic features of NASH^152^ but larger studies are needed especially to corroborate the effects on liver fibrosis.

Statins did not ameliorate the histological parameters in NASH.^153^ However, the treatment of dyslipidemia and of other features of the metabolic syndrome should be a primary target in patients with NAFLD.^16^ Statins may be safely prescribed and the risk of hepatotoxicity does not appear to be increased, even with NASH.^154^ Therapeutic targets are not specific for patients with NAFLD.

In hypertensive patients, inhibitors of the renin–angiotensin system should be the first choice agents, due to their potential antifibrogenic and insulin-sensitizing effects, although no robust data with hepatic histological endpoints are available.^155,156^

Several phase II clinical trials are ongoing to evaluate the effect of newer drugs with effects on different pathogenic pathways involved in NASH. These include anti-inflammatory, antifibrotic, antiapoptotic, and new metabolic modulator agents. FGF-19 or FGF-21 analogs, dual antagonist of chemokine receptors 2 and 5 and antilysyloxidase-like-2 monoclonal antibodies are examples of the diversity of drugs that are being tested for the treatment of NASH.^157^

It will be interesting to get data on genotypes and organokines’ profiles when interpreting the effect of different drugs.

Until effective drugs are available to treat NASH cirrhosis, liver transplantation use is expected to increase in the next 1 to 2 decades. It is important to know that the liver recipients are at risk for recurrence of NAFLD in the graft. A recent systematic review and meta-analysis compared survival and causes of death after liver transplantation for NASH and other etiologies.^158^ One-, three-, and five-year patient survivals were similar in NASH and non-NASH recipients; however, CVD events and sepsis were more frequent as causes of death in NASH recipients.

## Conclusion and future developments

The association between hepatic steatosis and CVD expands the spectrum of manifestations of unfavorable proatherogenic metabolic states, allowing the identification of new risk markers and the development of new therapeutic targets.

However, there lacks a clarification of the causal relationship between NAFLD and CVD, as well as of the complex interactions between genetic and environmental factors. The effectiveness of new therapeutic interventions also needs to be verified. Finally, the best cardiovascular risk score for patients with NAFLD needs to be further validated and vice versa, it is crucial to know if NAFLD/NASH should be included in CVD risk score panels.

## Acknowledgment

None.

## Conflicts of interest

The authors declare no conflicts of interest.

## Abbreviations

Abbreviations: ANGPTL-8 = Angiopoietin-like protein 8, CVD = Cardiovascular diseases, DPP-4 = Dipeptidyl peptidase-4, FGF-21 = Fibroblast growth factor 21, GLP-1 = Glucagon-like peptide 1, HDL = High-density lipoprotein, HIF-1α = Hypoxia-inducible factor 1- alpha, ^1^H-MRS = Proton magnetic resonance spectroscopy, IL = Interleukin, IMT = Intima-media thickness, LEAN = Liraglutide Efficacy and Action in Non-alcoholic steatohepatitis, LECT2 = Leukocyte cell derived chemotaxin 2, MetS = Metabolic syndrome, NAFLD = Nonalcoholic fatty liver disease, NASH = Nonalcoholic steatohepatitis, PNPLA3 = Patatin-like phospholipase domain containing-3, PPAR = Peroxisome proliferator-activated receptor, PUFAs = Polyunsaturated fatty acids, RCT = Randomized controlled trials, SEPP1 = Selenoprotein P , SGLT2 = Sodium–Glucose Co-Transporter 2, SHBG = Sex hormone-binding globulin, T2DM = Type 2 diabetes mellitus, TGF = Transforming growth factor, TM6SF2 = Transmembrane 6 superfamily member 2, TNF = Tumor necrosis factor, VLDL = Very low density lipoproteins.
